# Dynamic Changes in Systemic Inflammatory Indices Predict Residual High-Grade Lesions After Margin-Positive Cervical Conization: A Multicenter Retrospective Study

**DOI:** 10.3390/cancers18071114

**Published:** 2026-03-30

**Authors:** Gabriella Vajda, Kornél Lakatos, Lotti Lőczi, Balázs Vida, Nándor Ács, Barbara Sebők, Verita Szabó, Ádám Tabányi, Balázs Lintner, Richárd Tóth, Márton Keszthelyi

**Affiliations:** 1 Department of Obstetrics and Gynecology, Semmelweis University, 1082 Budapest, Hungary; vajda.gabriella@stud.semmelweis.hu (G.V.); loczi.lotti@semmelweis.hu (L.L.); vida.balazs.lajos@semmelweis.hu (B.V.); acs.nandor@semmelweis.hu (N.Á.); szabo.verita@gmail.com (V.S.); tabanyi.adam@semmelweis.hu (Á.T.); lintner.balazs.zoltan@semmelweis.hu (B.L.); toth.richard@semmelweis.hu (R.T.); 2 Maternity Obstetrics and Gynecology Private Clinic, 1126 Budapest, Hungary; 3 Faculty of Health and Sport Sciences, Széchenyi István University of Győr, 9026 Győr, Hungary; kornel.lakatos88@gmail.com; 4 Workgroup of Research Management, Doctoral School, Semmelweis University, 1085 Budapest, Hungary; sebok.barbara@semmelweis.hu

**Keywords:** cervical cancer, Platelet to Lymphocyte Ratio (PLR), systemic inflammation response index (SIRI), systemic immune inflammation index (SII), recognition, prognostic marker

## Abstract

**Background/Objectives**: Cervical cancer remains a global health burden. The loop electrosurgical excision procedure (LEEP) is effective for cervical intraepithelial neoplasia. Positive margins often complicate decisions about repeat conization. HPV testing is standard in post-treatment surveillance, but its limited specificity shows the need for additional, cost-effective biomarkers. This study evaluated whether changes in systemic inflammatory indices—platelet-to-lymphocyte ratio (PLR), systemic inflammation response index (SIRI), and systemic immune-inflammation index (SII)—can predict residual high-grade lesions after incomplete excision. **Methods**: This multicenter retrospective study included 125 patients who underwent repeat surgery after LEEP due to positive margins. Changes in preoperative inflammatory indices (ΔPLR, ΔSIRI, ΔSII) between the first and second procedures were analyzed by the histopathological findings of the second surgery. Group differences were assessed using the Mann–Whitney U test, and receiver operating characteristic (ROC) analysis was used to evaluate discriminatory performance. **Results**: Significant differences were found in ΔPLR (*p* = 0.032) and ΔSII (*p* = 0.048) between patients with and without residual high-grade lesions or invasive cancer. ΔSIRI showed borderline significance (*p* = 0.050). For invasive cancer alone, ΔSIRI was significantly associated with malignancy (*p* = 0.035). ROC analysis showed modest predictive performance (AUC ≈ 0.60). **Conclusions**: Dynamic changes in PLR, SIRI, and SII may be as inexpensive adjunct biomarkers to support risk stratification after incomplete LEEP and can complement HPV testing in certain clinical settings.

## Simple Summary

Cervical cancer remains a major global health burden. The loop electrosurgical excision procedure (LEEP) is an effective treatment for cervical intraepithelial neoplasia. However, positive surgical margins often complicate the decision on repeat conization. Systemic inflammatory markers such as PLR, SIRI, and SII have already been shown to effectively and cheaply predict histopathological outcomes after LEEP in cervical carcinoma. In cases of incomplete excision, though, their value for assessing residual dysplasia has not yet been explored. This study aims to determine whether changes in these indices between the first and second procedures can predict the presence of residual high-grade lesions or invasive cancer. The findings show modest but significant predictive performance. These readily available blood-based markers may complement current surveillance strategies, especially in low-resource settings. They may also support more individualized, evidence-based postoperative decisions, while encouraging further validation studies.

## 1. Introduction

Cervical cancer is the fourth most common malignancy among women worldwide [1] and the second most frequently diagnosed gynecological cancer [2]. In 2022, about 662,000 new cases and nearly 349,000 deaths were reported globally [3]. The incidence and mortality are much higher in low- and middle-income countries [4], but the disease remains a significant public health problem in high-income countries too [5]. Cervical cancer is considered entirely preventable based on current medical knowledge [6]. Its well-characterized pathomechanism supports this: persistent infection with high-risk human papillomavirus (HPV) is the main cause, and accounts for over 95% of cases [7]. The cancer develops through defined precursor lesions, progressing through stages of cervical intraepithelial neoplasia (CIN1–CIN3), which can be treated effectively when detected early [8]. The World Health Organization estimates that widespread HPV vaccination combined with effective population-based screening could eliminate the disease within decades [9].

Traditionally, cervical cancer screening has relied on cytological evaluation, most commonly performed using the Papanicolaou (Pap) test [10]. Abnormal cytological findings prompt further diagnostic assessment via colposcopic examination, with targeted biopsies of suspicious areas obtained for histopathological confirmation and to guide subsequent clinical management. Among therapeutic approaches, the loop electrosurgical excision procedure (LEEP) conization is widely used to treat CIN because it provides both a definitive diagnosis and excision of premalignant lesions.

LEEP conization aims to remove all dysplastic epithelium, but incomplete excision with positive surgical margins occurs in about a quarter to a third of cases [11]. Positive margins raise the risk of disease recurrence [7,12]. In these cases, clinicians must weigh the benefits and risks of repeat surgery, including re-conization, trachelectomy, or hysterectomy. Fertility preferences, cytological and histopathological findings, HPV status, and margin details should be considered [13]. HPV testing after surgery provides key guidance for decisions [8]. However, its low specificity [14] suggests a need for extra biomarkers in risk stratification.

Conventional inflammatory markers, including the neutrophil-to-lymphocyte ratio (NLR), lymphocyte-to-monocyte ratio (LMR), and platelet-to-lymphocyte ratio (PLR), are associated with the development of cervical cancer. They are considered indicators of systemic inflammation and immune dysregulation. Among these, PLR may reflect both increased thrombopoietic activity and lower lymphocyte counts. This makes PLR a marker that integrates tumor-promoting inflammation and reduced antitumor immune surveillance. High platelet counts are often associated with angiogenesis and tumor progression. Lymphopenia suggests compromised antitumor immune function [10,15]. Composite biomarkers such as the Systemic Immune-Inflammation Index (SII) and the Systemic Inflammation Response Index (SIRI) have recently received attention for their prognostic value. These indices use several leukocyte types and platelet counts. They show both pro-tumor inflammation and antitumor immune capacity. Higher PLR, SII, and SIRI have been found in patients with invasive carcinoma undergoing LEEP [10,16].

Therefore, the present study aimed to evaluate whether these biomarkers can be used not only to predict histopathological outcomes after LEEP conization, but also to support risk stratification in cases of incomplete excision.

## 2. Materials and Methods

### 2.1. Patients

**Figure 1:**
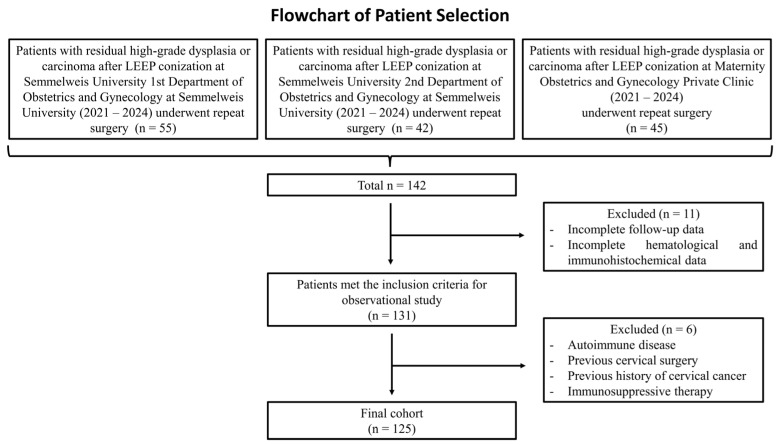
Patient selection flowchart. The figure illustrates the stepwise process for selecting study participants. Beginning with the initial cohort of individuals who underwent cervical conization and repeat conization, a series of clinical, histological, and data availability criteria were applied, resulting in the final study population.

This multicenter retrospective observational study evaluated a cohort of 125 patients enrolled in the SCOPE Study (Semmelweis University Conization and Inflammation Outcomes with Predictive Evaluation). Medical records from 2021 to 2024 were systematically reviewed (Figure 1).

#### Inclusion and Exclusion Criteria

An initial cohort of 142 patients who underwent cervical conization at three gynecological centers in Budapest—the 1st and 2nd Departments of Obstetrics and Gynecology of Semmelweis University and the Maternity Obstetrics and Gynecology Private Clinic—was screened. Eligible patients had undergone conization for cervical dysplasia with histopathological evidence of incomplete excision of either a high-grade lesion or invasive cancer, necessitating a second surgical intervention. The second procedure most commonly consisted of repeat conization; however, in selected cases, hysterectomy or trachelectomy was performed (Table 1).

Of the 142 initially identified patients, 11 were excluded due to incomplete follow-up or missing hematological parameters required to calculate ΔPLR, ΔSIRI, and ΔSII (e.g., missing neutrophil, lymphocyte, monocyte, or platelet counts), leaving 131 patients who met the inclusion criteria for the observational study. An additional 6 patients were excluded based on predefined clinical criteria, including autoimmune disease, prior cervical surgery, history of cervical cancer, or ongoing immunosuppressive therapy.

Notably, post-conization HPV genotyping data were not available for this cohort. According to ASCCP guidelines [17], the optimal timing for post-treatment HPV testing is six months after surgery. In our study, however, patients chose to undergo repeat surgery as soon as possible after the primary conization, rather than waiting for the recommended interval, precluding guideline-compliant post-conization HPV testing.

After application of these criteria, 125 patients were included in the final analysis. The resulting dataset encompassed sociodemographic characteristics, gynecological and obstetric history, clinical parameters, and laboratory findings, enabling a comprehensive assessment of patient outcomes following conization.

### 2.2. Characteristics

The sociodemographic data collected included patients’ age at the time of surgery (calculated as the year of surgery minus the year of birth), body mass index (BMI), laboratory parameters, cytology results, conization and repeat-surgery outcomes, and HPV status.

All laboratory assessments were conducted within 1 month prior to surgery in laboratories accredited by the National Accreditation Authority of Hungary, thereby ensuring compliance with recognized quality and accreditation standards. Laboratory analyses primarily focused on biomarkers of systemic inflammation, including neutrophil, monocyte, lymphocyte, and platelet counts. From these values, PLR, SIRI, and SII were calculated. 

All laboratory tests were performed within 1 month of both the initial conization and the second surgical procedure to ensure clinical relevance and temporal proximity to each intervention.

#### 2.2.1. Calculation of Inflammatory Indices

Systemic inflammatory indices were calculated from routine hematological parameters. Specifically, PLR, SIRI, and SII were derived using standard formulas:

$$PLR=\frac{Platelet\;count\;\left(\frac{G}{L}\right)\;}{Lymphocyte\;count\;(\frac{G}{L})}$$

$$SII=\frac{Platelet\;count\;\left(\frac{G}{L}\right)*Neutrophil\;granulocyte\;count\;(\frac{G}{L})\;}{Lymphocyte\;count\;(\frac{G}{L})}$$

$$SIRI=\frac{Neutrophil\;granulocyte\;count\;\left(\frac{G}{L}\right)*Monocyte\;count\;(\frac{G}{L})}{Lymphocyte\;count\;(\frac{G}{L})}$$

Dynamic changes in inflammatory markers (ΔPLR, ΔSIRI, ΔSII) were calculated as the difference between values measured prior to the second surgical procedure and those measured prior to the first conization:

Δ = value before second surgery − value before first surgery.

Thus, positive values indicate an increase in the marker level between the two procedures, while negative values indicate a decrease.

#### 2.2.2. Cervical Dysplasia Classification

Cervical dysplasia was assessed using cervical cytology results. Based on these findings, patients were categorized into four groups: Grade 1 (negative for intraepithelial lesion or malignancy), Grade 2 (low-grade squamous intraepithelial lesion [LSIL] and atypical squamous cells of undetermined significance [ASC-US]), Grade 3 (high-grade squamous intraepithelial lesion [HSIL], atypical squamous cells—cannot exclude HSIL [ASC-H], atypical glandular cells [AGC], and adenocarcinoma in situ [AIS]), and Grade 4 (invasive cervical cancer). Surgical data included histopathological evaluations of conization and repeat-surgery specimens, which were classified using the same four-tier system. This unified grading approach was implemented to align cytological and histological categories with similar clinical significance and management strategies, reducing diagnostic variability and minimizing the risk of underpowered statistical analyses in smaller subgroups. This approach follows current recommendations advocating integrated, risk-based categorization given the overlapping morphological features in cervical cytopathology. It is intended to ensure analytical consistency and does not replace standard clinical grading [18].

Ethical approval for this study was obtained from the Institutional Review Board of Semmelweis University (SE RKEB: 195/2024), in accordance with international ethical standards and institutional regulations governing patient confidentiality and data protection. The ethical approval was granted on 1 October 2024. 

### 2.3. Data Management

For this multicenter retrospective study, patient information was systematically collected and entered into a purpose-built electronic database for the SCOPE study. The database contained detailed sociodemographic, clinical, and laboratory data. Prior to statistical analysis, data quality was carefully evaluated using structured validation procedures, including manual and automated checks for inconsistencies and automated outlier detection via box plots to identify extreme values that might affect the results. Missing data were handled in accordance with predefined protocols to ensure the dataset’s completeness and reliability, preserving its integrity for subsequent analyses.

### 2.4. Statistical Analysis

Statistical analyses were performed using IBM SPSS Statistics for Windows, version 25.0 (Released 2017, IBM Corp., Armonk, NY, USA). Continuous variables were summarized using the mean, standard deviation, median, and range, while categorical variables were expressed as frequencies and percentages.

The normality of the laboratory parameters and indices—platelet-to-lymphocyte ratio (PLR), systemic inflammation response index (SIRI), and systemic immune-inflammation index (SII)—was assessed using the Shapiro–Wilk and Kolmogorov–Smirnov tests. As these variables did not follow a normal distribution, non-parametric methods were applied in subsequent analyses.

The accuracy of cytological screening was assessed using the McNemar test. Comparisons between groups were performed using the Mann–Whitney U test to evaluate differences in laboratory parameters and other variables, with particular focus on dynamic changes in PLR, SIRI, and SII according to the presence of high-grade squamous intraepithelial lesions (HSILs) or invasive cancer identified in the histopathological findings of the second surgical procedure. 

Receiver operating characteristic (ROC) curve analysis was conducted to evaluate the ability of dynamic changes in PLR, SIRI, and SII to discriminate between high-grade lesions or invasive cervical cancer and low-grade lesions or negative histological findings. The area under the ROC curve (AUC) was calculated to quantify the discriminatory performance of each marker. Optimal cutoff values for ΔPLR, ΔSIRI, and ΔSII were derived from ROC analysis using the Youden index and the cutoff nearest the top-left point of the curve.

## 3. Results

### 3.1. Patient Characteristics

Table 2 summarizes the baseline characteristics of the study population. A total of 125 conization procedures were included in the analysis; all cases required a second surgical intervention due to incomplete excision of dysplastic lesions. The median age was 42 years, and the median body mass index was 23.88 kg/m^2^. HPV status was available for 75 patients, of whom 73 (97.3%) were HPV-positive. Cervical cancer screening results were available for 120 patients. They were categorized into four groups: Grade I (negative) in 1 patient (0.83%), Grade II (LSIL, ASC-US) in 20 patients (16.7%), Grade III (HSIL, ASC-H, AGC, AIS) in 94 patients (78.3%), and Grade IV (cancer) in 5 patients (4.17%). Histopathological findings from the first conization and second surgical procedure were classified using the same four-grade system. For the first conization, only patients requiring repeat surgery due to high-grade lesions or cancer were included; 104 patients (83.2%) were classified as Grade III (HSIL, ASC-H, AGC, AIS) and 21 patients (16.8%) as Grade IV (cancer). Following the second surgery, histopathological evaluation revealed Grade I (negative) findings in 56 patients (44.8%), Grade II (LSIL, ASC-US) in 8 patients (6.4%), Grade III (HSIL, ASC-H, AGC, AIS) in 46 patients (36.8%), and Grade IV (cancer) in 15 patients (12.0%).

The median time interval between the first and second conization was 64.5 days (IQR: 50.5–100.3), with no statistically significant difference observed between the HSIL+ and Negative/LSIL groups (Mann–Whitney U test, *p* = 0.336).

Concerning the inflammatory markers calculated from the laboratory parameters analyzed in this study, prior to the first conization, the median PLR was 8.77 (IQR: 6.72–11.00), the median SIRI was 0.96 (IQR: 0.61–1.40), and the median SII was 505 (IQR: 368–680). Prior to the second surgery, the corresponding median values were 8.45 (IQR, 6.43–10.25) for PLR, 0.84 (IQR, 0.62–1.25) for SIRI, and 479 (IQR, 348–657) for SII (Table 3).

### 3.2. Diagnostic Accuracy of Cytological Screening

**Figure 2:**
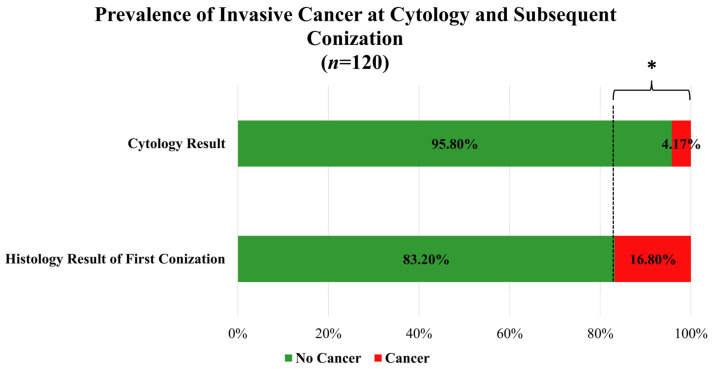
Proportion of invasive cancer detected by cytology and first conization histology. Bars represent the percentage of patients with and without invasive cancer in each diagnostic modality. An asterisk (*) denotes statistically significant differences between groups. Statistical comparison was performed using the McNemar test on paired observations (*n* = 120), *p* = 0.001. Sensitivity, specificity, positive predictive value (PPV), and negative predictive value (NPV) of cytology relative to conization histology are indicated.

In 120 cases, data were available for both cytological examination and histopathological examination of the first conization. Comparison of these modalities revealed both statistically and clinically relevant discordance. Invasive cancer was detected significantly more frequently at first conization than by cytology. While cytology identified invasive cancer in 5 of 120 patients (4.2%), histological examination of the first conization revealed invasive disease in 21 of 125 patients (16.8%). Among patients without invasive cancer on cytology, 16 cases were upgraded to invasive cancer at first conization, whereas only 2 cases showed the opposite change. This difference was statistically significant (McNemar test, exact *p* = 0.001; *n* = 120 paired observations). Based on the paired data, cytology’s sensitivity for detecting invasive cancer was 15.8%, while its specificity was 98.0%. The positive predictive value (PPV) was 60.0%, and the negative predictive value (NPV) was 86.1%. (Figure 2).

### 3.3. Relationship Between Laboratory Biomarkers and Histopathological Results of Second Surgery

#### 3.3.1. Presence of High Grade Lesions or Invasive Cancer (HSIL+)

**Figure 3:**
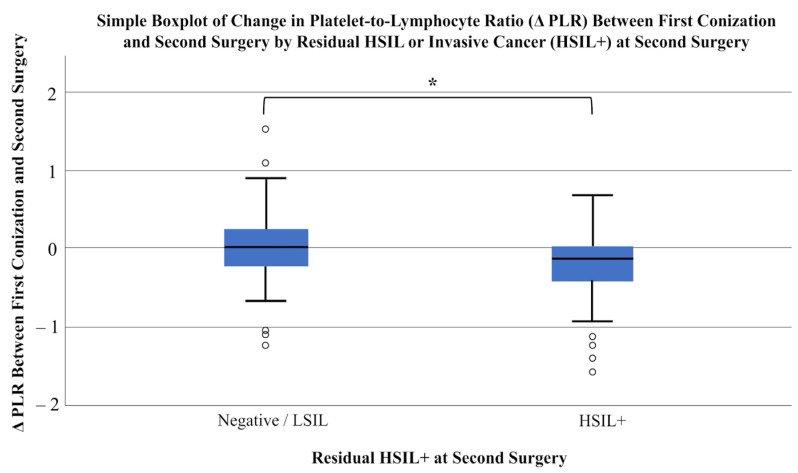
Simple Boxplot of change in Platelet-to-Lymphocyte Ratio (ΔPLR) between first conization and second surgery by residual HSIL or invasive cancer (HSIL+) at second surgery. Patients were divided into Negative/LSIL (*n* = 64) and HSIL+ (*n* = 61) groups based on histopathology of the second surgery. Statistical comparison was performed using the Mann–Whitney U test (*p* = 0.032). An asterisk (*) denotes statistically significant differences between groups.

**Figure 4:**
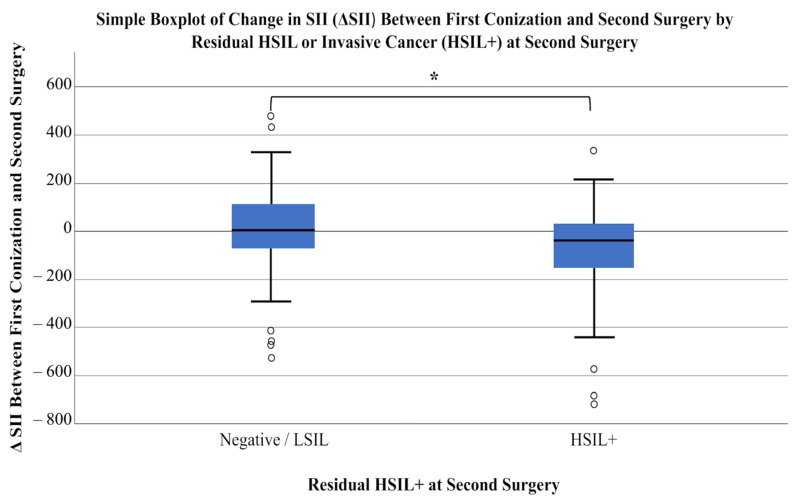
Simple Boxplot of change in SII (ΔSII) between first conization and second surgery by residual HSIL or invasive cancer (HSIL+) at second surgery. Circles indicate outliers, which were excluded to better display the data. Patients were divided into Negative/LSIL (*n* = 64) and HSIL+ (*n* = 61) groups based on histopathology of the second surgery. Statistical comparison was performed using the Mann–Whitney U test (*p* = 0.048). An asterisk (*) denotes statistically significant differences between groups.

**Figure 5:**
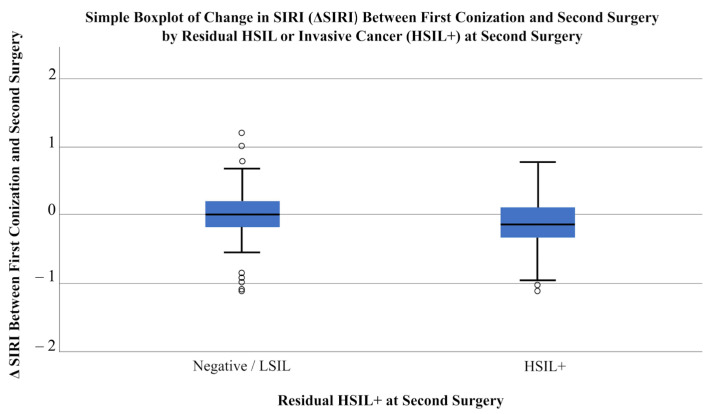
Simple Boxplot of change in SIRI (ΔSIRI) between first conization and second surgery by residual HSIL or invasive cancer (HSIL+) at second surgery. Circles indicate outliers, which were excluded to display the data distribution better. Patients were divided into Negative/LSIL (*n* = 64) and HSIL+ (*n* = 61) groups based on histopathology of the second surgery. Statistical comparison was performed using the Mann–Whitney U test (*p* = 0.050).

First, patients were divided into two groups based on histopathological results from the second surgery. The first group included patients with negative or low-grade lesions (Grades I and II), while the second group comprised those with high-grade lesions or invasive cancer (Grades III and IV). A total of 64 patients were classified as Negative/LSIL, and 61 as HSIL+. Using the Mann–Whitney U test, dynamic changes in laboratory parameters were compared between these groups. Statistically significant differences were observed in the dynamic changes in PLR (*p* = 0.032) and SII (*p* = 0.048). Additionally, delta SIRI showed borderline statistical significance (*p* = 0.050) (Table 4; Figure 3, Figure 4 and Figure 5).

#### 3.3.2. Presence of Invasive Cancer

Subsequently, the study population was divided into two groups based on the presence of invasive cancer in the histopathological results of the second surgery. The non-cancer group included 110 patients, while 15 patients were classified as having cancer. Using the Mann–Whitney U test for this grouping, only the dynamic change in SIRI among the analyzed laboratory biomarkers reached statistical significance (*p* = 0.035) (Table 5).

### 3.4. Diagnostic Performance

**Figure 6:**
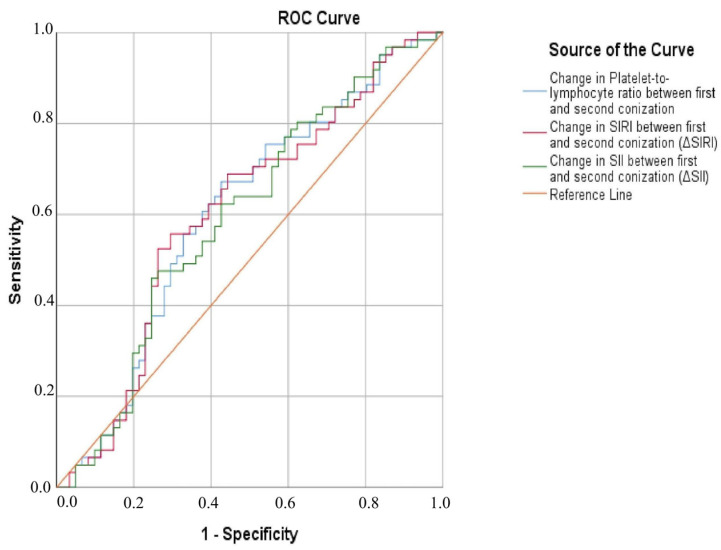
Receiver Operating Characteristic (ROC) Curve for ΔSIRI, ΔSII, and ΔPLR in predicting HSIL+ outcome of second surgery. Sensitivity (true positive rate) is plotted on the *y*-axis, while 1 − specificity (false positive rate) is on the *x*-axis. AUC: ΔPLR 0.603 (*p* = 0.050), ΔSIRI 0.603 (*p* = 0.050), ΔSII 0.594 (*p* = 0.073).

Receiver operating characteristic (ROC) analysis was performed to evaluate the diagnostic utility of changes in ΔSIRI, ΔSII, and ΔPLR for predicting HSIL+ outcomes at second surgery. Overall, the analysis indicated modest discriminatory performance across the evaluated inflammatory markers (Figure 6). The change in PLR between the first and second surgery achieved an area under the curve (AUC) of 0.603 (standard error [SE] = 0.052), with borderline statistical significance (*p* = 0.050) and a 95% confidence interval (CI) of 0.501–0.704. A comparable performance was observed for ΔSIRI, which yielded an identical AUC of 0.603 (SE = 0.052; *p* = 0.050; 95% CI: 0.501–0.705). In contrast, ΔSII showed a slightly lower discriminatory ability, with an AUC of 0.594 (SE = 0.052), and did not reach statistical significance (*p* = 0.073; 95% CI: 0.492–0.696).

Using the Youden index to determine optimal thresholds, the cut-off value for ΔPLR was −0.007 (HSIL+ if ≤ cut-off), corresponding to a sensitivity of 0.672 and a specificity of 0.574. This translated into a positive predictive value (PPV) of 0.612 and a negative predictive value (NPV) of 0.636, with likelihood ratios close to unity (+LR 1.58, −LR 0.57) and an overall accuracy of 0.623.

For ΔSIRI, the Youden-derived optimal cut-off was −0.136, yielding a lower sensitivity (0.525) but higher specificity (0.738) compared to ΔPLR. Notably, ΔSIRI demonstrated the highest positive likelihood ratio among the evaluated indices (+LR 2.00, −LR 0.64), with an overall accuracy of 0.631.

In the case of ΔSII, the optimal Youden cut-off was −73.729. Although specificity was moderate (0.754), sensitivity remained limited (0.459), resulting in likelihood ratios of +LR 1.87 and −LR 0.72 and an overall accuracy of 0.607 (Table 6).

## 4. Discussion

In this multicenter retrospective cohort study, we investigated women who underwent repeat surgery following LEEP conization with incomplete excision of the lesion. Our primary objective was to determine whether dynamic changes in CBC-derived inflammatory indices—specifically, PLR, SIRI, and SII—could predict the presence of residual high-grade dysplasia or invasive carcinoma in the histopathological specimen obtained at the second procedure. Both ΔPLR and ΔSII were significant predictors of residual high-grade dysplasia or invasive carcinoma, whereas ΔSIRI showed a borderline association. When the analysis was restricted to invasive carcinoma alone, ΔSIRI showed a statistically significant association.

Management after margin-positive conization remains clinically challenging because the decision to proceed with further surgical management—re-conization, trachelectomy, or hysterectomy—must be individualized based on clinicopathological factors and reproductive considerations. Although HPV genotyping is currently the most widely accepted test-of-cure strategy in this setting, its application is limited by substantial heterogeneity in assay types, testing intervals, number of post-treatment assessments, and duration of follow-up [19]. Moreover, HPV-based approaches require effective secondary triage to appropriately manage high-risk results and avoid unnecessary diagnostic escalation, particularly in resource-constrained settings [11]. Overall, these constraints indicate that complementary, cost-effective, and easily standardizable biomarkers may help refine post-treatment risk stratification after incomplete excision, particularly where complex HPV-based algorithms are difficult to implement. In our cohort, the HPV issue was not theoretical but directly influenced management. Although ASCCP recommends HPV testing about 6 months after excision, many patients chose earlier repeat surgery due to involved margins and the need for definitive treatment. As a result, guideline-timed HPV test-of-cure was unavailable, and re-intervention decisions relied on histopathology, cytology/colposcopy, and clinical judgment. This highlights the clinical relevance of our study: when timely HPV testing is unavailable or limited, low-cost, accessible biomarkers may help refine risk assessment.

At the same time, this also represents an important limitation. Persistent high-risk HPV infection is the most robust predictor of residual or recurrent cervical disease; therefore, the absence of HPV follow-up precludes direct comparison between inflammatory markers and HPV-based risk stratification and limits the interpretability of our findings. It remains unclear whether the observed associations are independent of HPV persistence or may partially reflect underlying viral status. Accordingly, these results should be interpreted as complementary to, rather than a substitute for, HPV-based surveillance, and primarily applicable to clinical scenarios in which HPV testing is unavailable, delayed, or not feasible.

The exploration of complete blood count (CBC)-derived biomarkers in oncology is based on the recognition that chronic, low-grade inflammation constitutes a fundamental component of the tumor microenvironment [12,20]. Accumulating evidence supports the prognostic significance of NLR, PLR, and LMR across a broad spectrum of malignancies, including osteosarcoma [21], non-small cell lung cancer [12], and endometrial cancer [22]. Among these markers, PLR warrants particular attention, as it may more comprehensively capture the interplay between enhanced thrombopoietic activity and reduced lymphocyte counts, thereby reflecting both tumor-promoting inflammation and impaired antitumor immune surveillance. In cervical cancer, the prognostic relevance of PLR has also been demonstrated; Kalas et al. reported a significant association between elevated PLR levels and invasive disease [10]. Building on these findings, we analyzed the dynamic change in PLR (ΔPLR) between the initial conization and subsequent surgery. When the outcome was restricted to invasive cancer at the second procedure, ΔPLR did not reach statistical significance, likely reflecting the limited number of invasive cases. However, when residual high-grade dysplasia and invasive cancer were evaluated together (HSIL+), ΔPLR emerged as a significant predictor. These results extend the observations of Kalas et al., suggesting that PLR, as an integrative marker of tumor-promoting inflammation and impaired antitumor immunity, may serve not only as a predictor of invasive disease but also as a robust indicator of residual high-grade dysplasia.

Beyond two-parameter indices such as NLR, PLR, and LMR, increasing attention has been directed toward composite markers including SII and SIRI. These indices integrate multiple hematologic parameters—neutrophils, lymphocytes, and platelets for SII, and neutrophils, lymphocytes, and monocytes for SIRI—thereby providing a more comprehensive reflection of systemic inflammatory status and immune balance. In contrast to simpler ratios that capture more limited aspects of inflammation (e.g., neutrophil predominance over adaptive immunity), SII and SIRI simultaneously reflect tumor-promoting inflammatory activity and impaired antitumor immune surveillance [16,23,24]. This broader integration may enhance their prognostic relevance by more accurately characterizing the complex interplay between pro-tumor inflammation and immune suppression. Consistent with this rationale, the predictive value of SII and SIRI has been extensively investigated in oncology. Multiple studies have demonstrated a significant association between SIRI and both progression-free and overall survival in nasopharyngeal carcinoma [25] and soft tissue sarcoma [23], whereas the prognostic relevance of SII has been confirmed in colorectal cancer [26], esophageal squamous cell carcinoma [27], and oral squamous cell carcinoma [28]. Moreover, higher SIRI and SII levels have been shown to be significant predictors of cervical dysplasia and malignancy; Keszthelyi et al. demonstrated in a retrospective study including 344 patients undergoing LEEP conization that higher SII and SIRI values were significantly associated with high-grade lesions and invasive carcinoma [16]. These findings are in line with our results: ΔSIRI emerged as a significant predictor of invasive cancer and showed a threshold-dependent association when residual high-grade lesions and invasive cancer were analyzed together (HSIL+), whereas ΔSII was significant for predicting HSIL+ outcomes. Collectively, these observations support the utility of these indices, which comprehensively reflect systemic immune status, not only for predicting invasive disease but also for identifying high-grade, near-invasive lesions.

While these findings are encouraging, the markers’ diagnostic performance was modest. ROC analyses demonstrated only borderline discriminative ability for ΔPLR and ΔSIRI (AUC values of approximately 0.60), whereas ΔSII did not reach statistical significance in ROC testing. From a clinical standpoint, this level of accuracy is generally considered limited and insufficient for reliable individual risk prediction. Accordingly, these indices are not suitable as standalone diagnostic tools and should not be interpreted as replacements for HPV-based surveillance, which remains the most sensitive and reliable method for detecting residual or recurrent disease. Although statistically significant, the observed associations have limited clinical applicability when considered in isolation. Instead, the most plausible role of these markers is as adjunctive tools that may provide incremental information within a broader clinical framework. In particular, they may contribute to risk stratification when interpreted alongside histopathological findings, clinical parameters, and—when available—HPV status, especially in scenarios where HPV test-of-cure is unavailable, delayed, or operationally difficult.

Our findings also underscore the limits of cytology for detecting invasive disease. While a 2016 meta-analysis reported 74% sensitivity for detecting post-treatment high-grade lesions [29], invasive cancer was identified far more frequently by first-conization histology than by cytology.

This discrepancy reinforces why post-treatment surveillance increasingly relies on HPV testing, which generally achieves higher sensitivity and negative predictive value than cytology for detecting residual/recurrent HSIL. From that perspective, the current study should be interpreted as addressing a practical gap: in the absence of timely HPV test-of-cure, can readily available host-response markers help stratify risk among margin-positive patients? ΔPLR exhibited the highest sensitivity among our markers (67.2%), suggesting some ability to identify at-risk patients. Yet, its NPV (63.6%) remained far below that of HPV testing, with reported sensitivities ranging from 69% to 92% and NPVs between 97% and 99%, limiting confidence in ruling out residual disease [29,30]. Conversely, ΔSIRI demonstrated the highest specificity (73.8%) and PPV (66.7%), with favorable likelihood ratios (+LR 2.00, −LR 0.64; accuracy 0.631), indicating a reasonable capacity to identify true residual disease. This is consistent with broader oncologic evidence, where dynamic changes in inflammatory or tumor markers often carry prognostic value [31,32,33]; yet, unlike studies in systemic or advanced malignancies—where increases in markers predict poor outcomes—we observed that decreases in ΔSII and ΔSIRI correlated with residual cervical disease.

This seemingly paradoxical finding—namely, that decreases in inflammatory indices were associated with residual disease—may be explained by the specific clinical context and the dynamic nature of the markers evaluated. In contrast to studies assessing baseline inflammatory status in untreated malignancies, our analysis captures changes occurring between two surgical interventions. The initial conization itself represents a form of tissue injury that can induce a transient systemic inflammatory response, characterized by increased neutrophil and platelet counts as part of the acute-phase reaction [34,35]. In patients without residual disease, this post-surgical inflammatory activation may persist longer or resolve more gradually.

Conversely, in patients with residual lesions, persistent tumor tissue may promote a more immunosuppressive microenvironment, characterized by impaired lymphocyte-mediated antitumor immunity and altered myeloid cell dynamics. Tumor-associated immune evasion mechanisms—including the expansion of regulatory T cells and myeloid-derived suppressor cells—have been shown to suppress effective systemic immune responses while maintaining local tumor-promoting inflammation [36,37,38]. This immunosuppressive shift may lead to relatively lower circulating inflammatory indices such as SII and SIRI, which integrate neutrophil, lymphocyte, and platelet or monocyte counts.

In addition, accumulating evidence suggests that dynamic changes in inflammatory markers may carry distinct biological significance compared to static baseline values. While elevated baseline indices are generally associated with tumor burden and poor prognosis, declining levels over time—particularly in the presence of persistent disease—may reflect immune exhaustion or inadequate host response rather than true disease resolution. Similar observations have been reported in other malignancies, in which impaired lymphocyte recovery or declining inflammatory ratios were associated with reduced immune competence and adverse outcomes [39,40].

Taken together, these findings highlight that, in the post-conization setting, decreases in systemic inflammatory indices should be interpreted contextually and may paradoxically indicate an insufficient systemic immune response to residual disease rather than clinical improvement.

Collectively, these findings reinforce that HPV testing remains the most reliable method for post-conization surveillance, providing the highest sensitivity and the greatest assurance of disease absence. Inflammatory markers, while informative, appear most useful as adjuncts to refine risk stratification or to identify patients warranting closer follow-up, rather than as substitutes for established molecular surveillance strategies. Future studies integrating dynamic inflammatory indices with HPV testing may clarify whether these markers can improve individualized post-treatment management and early detection of residual or recurrent disease.

### 4.1. Strengths and Limitations

The principal strengths of the present investigation lie in its multicenter design, robust statistical methods, and diverse analytical techniques. These included the Mann–Whitney U test, receiver operating characteristic (ROC) curve analysis, and the determination of optimal cut-off values using both the Youden index and the closest top-left point method.

Nevertheless, the retrospective nature of the study entails several inherent limitations, most notably the risk of selection and information bias. In contrast to randomized controlled trials, the absence of randomization and prospective data collection may restrict the external validity and generalizability of the findings.

The relatively small sample size (*n* = 125) may limit the generalizability of our results. In addition, the analysis was restricted to patients with complete clinical and laboratory data; therefore, excluding cases with missing hematological or follow-up information may have introduced selection bias by favoring individuals with more comprehensive clinical documentation and closer postoperative surveillance.

Furthermore, although individuals with conditions known to influence PLR, SIRI, and SII were excluded, residual confounding cannot be excluded, as potentially relevant factors—such as comorbidities and concomitant medications—were not comprehensively evaluated.

Detailed surgical parameters of the initial conization procedure—such as cone depth, width, excision volume, margin location, multifocality, and glandular involvement—were not consistently available in the dataset and therefore could not be included in the analysis. In addition, key pathological characteristics of the cervical lesions, including multifocality, glandular involvement, localization of positive margins (endocervical vs. ectocervical), and lesion size or extent, were also incomplete.

These variables are well-established determinants of residual disease and play a significant role in post-treatment clinical decision-making. Their absence represents a potential source of confounding; consequently, the observed associations between inflammatory markers and residual lesions may partly reflect underlying differences in surgical technique and lesion characteristics rather than solely systemic inflammatory status.

Another limitation of our study is the lack of complete HPV data. Post-treatment HPV follow-up was often unavailable because many patients underwent early repeat surgery after margin-positive conization, preventing assessment of HPV persistence. In addition, preoperative HPV status was documented in only 75 of 125 cases. This reflects the retrospective, multicenter design and variability in clinical practice, but may introduce selection bias. Furthermore, the high proportion of HPV-positive cases limited subgroup analyses and prevented adjustment for HPV status in our models. Therefore, the potential influence of HPV status on the observed associations between inflammatory markers and residual disease cannot be excluded.

A further limitation of our study is the heterogeneity of secondary surgical procedures, including repeat conization, trachelectomy, and hysterectomy. The choice of procedure was individualized based on clinical risk assessment, patient fertility desires, psychological factors, and overall health. While the primary outcome—residual lesion—was evaluated regardless of procedure type, this heterogeneity may have influenced the observed associations and should be considered when interpreting the results. The number of patients diagnosed with invasive cancer at the second procedure was relatively small (*n* = 15), reducing the statistical power of subgroup analyses and affecting the robustness of the corresponding comparisons. An additional limitation of this study is the absence of multivariable analysis. Although such modeling would be important to evaluate the independent predictive value of inflammatory markers, it was not feasible due to the relatively small sample size and the limited number of outcome events, which would increase the risk of model overfitting and unstable estimates. Furthermore, several clinically relevant confounding variables were not consistently available in this retrospective dataset, precluding the use of adequately adjusted models. Therefore, the present findings should be interpreted as exploratory, and future larger prospective studies are required to confirm these results using multivariable approaches.

Finally, we used a combined HSIL+ endpoint, encompassing both residual high-grade dysplasia and invasive carcinoma, to reflect clinically relevant post-conization management decisions and maintain sufficient statistical power given the low number of invasive cases; however, this composite endpoint represents a methodological limitation, as HSIL and invasive cancer are distinct pathological entities.

### 4.2. Implication for Practice

Given their low cost, widespread availability, and straightforward calculation from routine hematological parameters, PLR, SIRI, and SII may serve as adjunctive biomarkers in the evaluation of cervical lesions. Although these indices are not intended to replace cytological or histopathological assessment, they may provide additional support for postoperative risk stratification following incomplete excision by LEEP conization, particularly in healthcare settings where access to advanced diagnostic modalities is limited.

In clinical practice, dynamic changes in PLR, SIRI, and SII could contribute to more refined decision-making by helping to identify patients at increased risk of residual high-grade dysplasia or invasive carcinoma in the presence of positive surgical margins. Their incorporation into clinical assessment algorithms may enhance risk stratification and promote more efficient allocation of healthcare resources in both resource-rich and resource-constrained environments.

## 5. Conclusions

Substantial evidence supports the use of static PLR, SII, and SIRI as predictive biomarkers in cervical cancer, with proven prognostic value across various malignancies. However, in patients with margin-positive LEEP conization, the potential role of dynamic changes in these indices for risk stratification has not been explored. In our study, ΔPLR, ΔSIRI, and ΔSII were significantly associated with residual high-grade dysplasia or invasive carcinoma. Although their discriminatory performance was modest (ROC AUC ~0.60), these markers are not suitable as standalone predictors. Still, they may serve as supportive adjuncts, providing complementary information alongside clinical, histopathological, and HPV data, with HPV testing remaining the most sensitive and reliable method for post-treatment surveillance.

## Figures and Tables

**Table 1:** Distribution of patients according to the type of second surgical procedure performed following incomplete excision at initial conization.

Type of Second Surgical Procedure	Number of Patients
Re-Conization	78
Hysterectomy	44
Trachelectomy	3
Total	125

**Table 2:** Characteristics of the sample. Categorical parameters are presented as *n*. Continuous data are presented as median (interquartile range).

Characteristics (*n* = 125)	*N* (Range or %)
Total	125
Median age (years)	42 (35–48)
Median BMI (kg/m^2^)	23.88 (21.26–26.06)
HPV-status	75
Positive	73 (97.3%)
Negative	2 (2.7%)
Cytology results	120
Grade I	1 (0.83%)
Grade II	20 (16.7%)
Grade III	94 (78.3%)
Grade IV	5 (4.17%)
Primary conization results	
Grade III	104 (83.2%)
Grade IV	21 (16.8%)
Second surgery results	
Grade I	56 (44.8%)
Grade II	8 (6.4%)
Grade III	46 (36.8%)
Grade IV	15 (12.0%)

**Table 3:** Comparison of inflammatory markers (PLR, SIRI, and SII) calculated from laboratory parameters before the first and second surgical procedures.

Inflammatory Marker	Before the First Conization Median (IQR)	Before the Second Surgery Median (IQR)
PLR	8.77 (6.72–11.0)	8.45 (6.43–10.25)
SIRI	0.96 (0.61–1.40)	0.84 (0.62–1.25)
SII	505 (368–680)	479 (348–657)

**Table 4:** Mann–Whitney U test results comparing dynamic changes in laboratory biomarkers (ΔSIRI, ΔSII, and ΔPLR) between patients with and without high-grade lesions or invasive cancer at the second conization. *Z* = standardized test statistic; Asymptotic Significance (2-tailed) = *p*-value for the two-tailed test. * *p* < 0.05.

	Change in SIRI Between First and Second Surgery (ΔSIRI)	Change in SII Between First and Second Surgery (ΔSII)	Change in PLR Between First and Second Surgery (ΔPLR)
Mann–Whitney U	1477.0	1551.0	1519.0
Z	−1.964	−1.981	−2.139
Asymptotic Significance (2-tailed)	0.050	0.048 *	0.032 *

**Table 5:** Mann–Whitney U test results comparing the dynamic changes in the Systemic Inflammatory Response Index (ΔSIRI) between patients with and without invasive cancer at the second conization. Z = standardized test statistic; Asymptotic Significance (2-tailed) = *p*-value for the two-tailed test. * *p* < 0.05.

	Change in SIRI Between First and Second Surgery (ΔSIRI)
Mann–Whitney U	532.000
Z	−2.109
Asymptotic Significance (2-tailed)	0.035 *

**Table 6:** Cut-off values via the Youden index and the Closest Top Left method. The table shows the optimal cut-off values, determined using the Youden index and the Closest Top Left method, to assess the sensitivity and specificity of ΔPLR, ΔSIRI, and ΔSII.

	Cut-Off	Sensitivity	1 − Specificity	Positive Predictive Value (PPV)	Negative Predictive Value (NPV)	Positive Likelihood Ratio (+LR)	Negative Likelihood Ratio (−LR)	Accuracy
ΔPLR								
Youden-index	−0.007	0.672	0.426	0.612	0.636	1.577	0.571	0.623
Closest Top Left Method	−0.007	0.672	0.426	0.612	0.636	1.577	0.571	0.623
ΔSIRI								
Youden-index	−0.136	0.525	0.262	0.667	0.608	2.000	0.644	0.631
Closest Top Left Method	−0.095	0.557	0.295	0.654	0.614	1.889	0.628	0.631
ΔSII								
Youden-index	−73.729	0.459	0.246	0.651	0.582	1.867	0.717	0.607
Closest Top Left Method	−7.102	0.623	0.426	0.594	0.603	1.462	0.657	0.598

## Data Availability

The data supporting the findings of this study are available upon request from the corresponding author.

## Abbreviations

The following abbreviations are used in this manuscript:

+LR	Positive Likelihood Ratio
−LR	Negative Likelihood Ratio
AGC	Atypical Glandular Cells
AIS	Adenocarcinoma In Situ
ASC-H	Atypical Squamous Cells cannot exclude a high-grade lesion
ASC-US	Atypical Squamous Cells of Undetermined Significance
ASCCP	American Society for Colposcopy and Cervical Pathology
AUC	Area Under Curve
BMI	Body Mass Index
CBC	Complete Blood Count
CIN	Cervical Intraepithelial Neoplasia
CI	Confidence Interval
HPV	Human Papillomavirus
HSIL	High-grade Squamous Intraepithelial Lesion
IQR	Interquartile Range
LEEP	Loop Electrosurgical Excision Procedure
LMR	Lymphocyte-to-Monocyte Ratio
LSIL	Low-grade Squamous Intraepithelial Lesion
NLR	Neutrophil-to-Lymphocyte Ratio
NPV	Negative Predictive Value
PLR	Platelet-to-Lymphocyte Ratio
PPV	Positive Predictive Value
ROC	Receiver Operating Characteristic
SCOPE	Semmelweis University Conization and Inflammation Outcomes with Predictive Evaluation
SE	Standard Error
SII	Systemic Immune-Inflammation Index
SIRI	Systemic Inflammation Response Index
